# Supplementary material for: Lactobacilli and other gastrointestinal microbiota of Peromyscus leucopus, reservoir host for agents of Lyme disease and other zoonoses in North America
Source: PLoS One. 2020 Aug 20;15(8):e0231801. doi: 10.1371/journal.pone.0231801 (PMC7446861; doi:10.1371/journal.pone.0231801)
Supplement: S4 Table — (DOCX) [file pone.0231801.s008.docx]

| Table S4. Putative bacteriocins and associated transport proteins and immunity proteins of 3 *Lactobacillus* species of *Peromyscus leucopus* | | | | | | | | | |
| --- | --- | --- | --- | --- | --- | --- | --- | --- | --- |
| Species | Protein type | Activity | Motif | Replicon/  fragment | Accession | Start position | End position | Amino acids | Comment |
| *animalis* | Circularin_A | Class I bacteriocin | pfam09221 | Chromosome |  | 54877 | 55170 | 97 | Enterocin of *Enterococcus faecium* LC068607; head-to-tail cyclized peptide |
| *animalis* | Circularin_A | Class I bacteriocin | pfam09221 | Chromosome |  | 1781957 | 1782187 | 76 | Circular bacteriocin of *L. animalis* QCQ04638 (100% identity) |
| *johnsonii* | Bacteriocin_ABC (SunT) | Export | COG2274 | Contig 15 |  | 13 | 1863 | 615 | Export ABC transporter of *L. johnsonii* TGA95045 (98% identity) |
| *johnsonii* | EntA_Immun | Immunity | pfam08951 | Contig 15 |  | 2512 | 2796 | 94 | Bacteriocin immunity protein of *L. johnsonii* AZZ67109 (97% identity) |
| *johnsonii* | EntA_Immun | Immunity | pfam08951 | Contig 15 |  | 3822 | 4136 | 104 | Bacteriocin immunity protein of *L. johnsonii* AHA97049 (99% identity) |
| *johnsonii* | Helveticin_J | Class III bacteriocin | pfam17312 | Contig 25.33 |  | 29412 | 30470 | 352 | Hypothetical protein of *L. johnsonii* EEJ59115 (97% identity) |
| “peromyscii” | Helveticin_J | Class III bacteriocin | pfam17312 | Contig 1 |  | 843558 | 844523 | 321 | Bacteriocin of *L. helveticus* ANZ56396 (76% identity) |
| “peromyscii” | Helveticin_J | Class III bacteriocin | pfam17312 | Contig 1 |  | 848943 | 849980 | 345 | Bacteriocin of *L. helveticus* ABX26363 (48% identity) |
| “peromyscii” | Helveticin_J | Class III bacteriocin | pfam17312 | Contig 1 |  | 1631670 | 1632629 | 319 | Bacteriocin of L. crispatus KAA8780420 (63% identity) |
|  |  |  |  |  |  |  |  |  |  |
